# Surgical Outcomes of Collagen Patch Versus Fat Plug in the Repair of Tympanic Membrane Perforations: A Prospective Study

**DOI:** 10.7759/cureus.95825

**Published:** 2025-10-31

**Authors:** Rajeshwari Nachiyar, Rajasekar M K

**Affiliations:** 1 Otolaryngology - Head and Neck Surgery, Sree Balaji Medical College and Hospital, Chennai, IND

**Keywords:** collagen patch, fat plug, myringoplasty, otologic surgery, tympanic membrane perforation

## Abstract

Small perforations of the tympanic membrane (TM) remain a common concern in otology, with potential consequences such as recurrent infections, conductive hearing loss, and cholesteatoma formation if left untreated. Conventional grafting materials like temporalis fascia are widely accepted, but minimally invasive office-based alternative, including collagen patch and fat plug, are increasingly recognized for their efficacy and patient convenience. This prospective study was conducted at Sree Balaji Medical College and Hospital, Chrompet, Chennai, and included 46 patients aged 20-50 years with small central TM perforations. Participants were randomly assigned to undergo either collagen patch myringoplasty or fat plug myringoplasty, and outcomes were evaluated through otoscopic examinations and audiometric assessments at baseline and at one month and two months postoperatively. Both techniques demonstrated significant postoperative improvement in air-bone gap (p<0.0001). At two months, complete closure was achieved in 91.3% of patients in the fat plug group compared to 78.3% in the collagen patch group. Fat plug myringoplasty showed fewer failures, particularly in cases with traumatic and post-myringoplasty perforations, whereas infection and otomycosis during the healing phase were the primary causes of graft failure in both groups. While early healing rates were comparable, fat plug myringoplasty demonstrated slightly superior long-term outcomes. These findings suggest that both collagen patch and fat plug myringoplasty are effective, minimally invasive options for repairing small TM perforations. However, fat plug myringoplasty appears to offer higher closure rates and greater reliability in complex etiologies, supporting its value as a practical alternative in office-based otologic surgery.

## Introduction

The tympanic membrane (TM), or eardrum, is a thin trilaminar structure that plays a critical role in hearing and middle ear protection. It transmits acoustic vibrations from the external auditory canal to the ossicles while serving as a barrier against pathogens and foreign material. Anatomically, it consists of an outer epithelial layer, a fibrous middle layer of radial and circular collagen fibres, and an inner mucosal lining continuous with the middle ear cavity [1]. This architecture allows both vibration and durability.

Despite its resilience, TM is susceptible to perforation from various causes, including trauma, infections, barotrauma, and iatrogenic injury. Even small perforations may lead to conductive hearing loss, recurrent otorrhea, and, in chronic cases, acquired cholesteatoma formation [2,3]. Although many small perforations heal spontaneously, persistence is common when confounding factors such as chronic infection, diabetes, or immunosuppression interfere with healing [4].

Conventional repair techniques have relied on autologous grafts, most notably temporalis fascia, which provides high closure rates and favourable audiological outcomes [5]. However, harvesting fascia requires an additional surgical step, extending operative time and exposing patients to donor-site morbidity. This has led to increasing interest in less invasive, office-based alternatives, particularly for small perforations.

One such technique is fat plug myringoplasty, typically using ear lobule fat. The method is simple, minimally invasive, and often performed under local anaesthesia. Success rates range from 70% to 90%, though failures may occur due to fat resorption, graft displacement, or ongoing infection [6].

In parallel, advancement in biomaterials have introduced options such as paper patches, gel foam, and hyaluronic acid derivatives. More recently, engineered materials including silk fibroin, chitosan, and collagen scaffolds have been evaluated in tympanic membrane repair [7].

Among these, collagen patches are promising because collagen is a natural component of the TM’s fibrous layer. Collagen patches act as scaffolds for fibroblast migration and tissue regeneration, allowing perforation closure without donor-site harvesting [8]. However, concerns remain regarding durability, biodegradation, cost, and performance in infected ears.

Comparative data between these two minimally invasive methods remain limited. A recent prospective study demonstrated that both fat plug and collagen patch myringoplasty achieved significant improvement in closure rates and air-bone gap reduction, with fat plugs showing slightly higher success in traumatic and recurrent perforations [9]. Such findings highlight the need for further clinical evaluation of these techniques.

The present study was undertaken to compare the outcomes of collagen patch myringoplasty and fat plug myringoplasty in small TM perforations. Primary endpoints included anatomical closure rates, healing timeline, and audiometric improvement. Secondary objectives were to analyze etiology-specific success and identify causes of graft failure. By focusing on these two minimally invasive approaches, we aim to provide evidence to guide clinical decision making and improve patient outcomes in routine otologic practice.

## Materials and methods

Study design

This research was a prospective, comparative observational study evaluating the effectiveness of collagen patch versus fat plug myringoplasty in repairing small TM perforations. The study was conducted from November 2023 to November 2024 at the Department of Otorhinolaryngology, Sree Balaji Medical College and Hospital, Chennai. Standardized interventions, clinical assessments, and follow-ups ensured uniformity in patient evaluation and minimized bias.

Study setting

Patients were enrolled through the outpatient clinics, and procedures were performed in the minor operation theatre under local anaesthesia with strict aseptic precautions. Postoperative assessments were conducted in ear, nose and throat (ENT) outpatient clinics.

Study population

Patients aged 20-50 years with small central TM perforations were included after clinical and audiometric evaluation to rule out ossicular discontinuity or sensorineural hearing loss.

Sampling and sample size calculation

A purposive sampling technique was used. The sample size was calculated based on previous studies reporting closure rates of 70%-90% for small TM perforations repaired via fat plug or collagen patch techniques [10]. Using a two-tailed comparison, with 95% confidence and 80% power, 46 patients (23 in each group) were required.

Inclusion criteria

Patients eligible for inclusion were between 20 and 50 years of age and had a single, small central tympanic membrane perforation with a dry ear for at least three months. All patients had normal ossicular function and consented to a minimum follow-up period of two months.

Exclusion criteria

Patients with active ear discharge, marginal perforations, or cholesteatoma were excluded. Additional exclusion criteria included tympanosclerosis, Eustachian tube dysfunction, bilateral tympanic membrane perforations, prior failed tympanoplasty or surgery on the same ear, and the presence of sensorineural hearing loss or ossicular discontinuity.

Grouping and allocation

Randomization was performed using the sealed-envelope method by an independent ENT surgeon. Patients were divided into: Group A (collagen patch myringoplasty) and Group B (fat plug myringoplasty).

All procedures were performed by the same surgeon to reduce variability.

Preoperative assessment

All patients underwent a comprehensive ENT examination with otoendoscopy, pure tone audiometry to document thresholds and calculate the air-bone gap, and routine blood investigations. Written informed consent was obtained from all participants after explaining the procedure, its risks, benefits, and the follow-up protocol.

Surgical techniques

Collagen patch myringoplasty (Group A) was performed under local anesthesia in the outpatient department. The TM edges were freshened with a Rosen needle, and a sterile collagen sheet was placed over the perforation and supported with gelfoam. Postoperative care included antibiotic ear drops, analgesics, and standard ear precautions.

Fat plug myringoplasty (Group B) was performed under local anesthesia in the minor operation theatre. Fat was harvested from the ear lobule through a 1 cm incision, and the perforation edges were freshened. The fat plug was then inserted using the “cigarette roll” technique and packed with gelfoam. Postoperative care was identical to that of Group A. Both procedures lasted approximately 15-25 minutes and did not require general anesthesia.

Postoperative assessment and follow-up

Postoperative follow-up evaluations were carried out at standardized intervals. On day 7, early healing was assessed; on day 14, intermediate healing was documented. At one month, repeat audiometry and otoscopic examination were performed, and at two months, the final assessment of graft uptake and hearing improvement was conducted.

The primary outcomes were graft uptake at two months and hearing improvement, measured as a reduction in the air-bone gap. Secondary outcomes included the extent of healing at days 7 and 14, complications such as otomycosis, persistent discharge, or graft failure, and the time required for complete epithelialization.

Operational definitions

Complete graft uptake was defined as an intact tympanic membrane with no residual perforation at two months. Air-bone gap improvement was defined as the difference between preoperative and postoperative audiometric thresholds. Failure was considered as a persistent or recurrent perforation beyond one month. Complications referred to any deviation from expected healing, including otorrhea or granulation tissue formation.

Statistical analysis

Data were analyzed using SPSS version 9.0 (SPSS Inc., Chicago, IL). Descriptive statistics were expressed as mean, standard deviation, and proportions. The chi-square test was used to compare closure and complication rates, while the unpaired t-test was applied to analyze hearing outcomes (air-bone gap). A p-value of <0.05 was considered statistically significant.

Ethical considerations

Ethical clearance obtained from the Institutional Ethics Committee of Sree Balaji Medical College. The study adhered to the Declaration of Helsinki [11]. Confidentiality of patient data was maintained.

## Results

Demographic and baseline characteristics

A total of 46 patients were included, with 23 undergoing collagen patch myringoplasty (Group A) and 23 undergoing fat plug myringoplasty (Group B).

Age distribution

The mean age was 33.8±9.5 years in the collagen group and 30.0±10.6 years in the fat group (t=1.28., p=0.20). Age distribution across both groups is presented in Table 1. In the collagen group, eight patients (34.8%) were aged 20-30 years and 15 (65.2%) were aged 31-50 years. In the fat group, 11 patients (47.8%) were aged 20-30 years and 12 (52.2%) were aged 31-50 years. No statistically significant difference was observed.

**Table 1:** Age distribution of patients in collagen and fat plug myringoplasty groups. Values are expressed as N (%) and mean±SD. Independent t-test applied; results reported as t-value with p-value; p<0.05 considered statistically significant.

Age group (years)	Collagen (n=23)	Fat (n=23)	t-value	p-value
20-30	8 (34.8%)	11 (47.8%)	1.28	0.2
31-50	15 (65.2%)	12 (52.2%)	1.28	0.2
Mean±SD	33.8±9.5	30.0±10.6	1.28	0.2

Gender distribution

Both groups had identical gender representation: 12 patients (52.2%) were men and 11 (47.8%) were women in each group (Table 2). Chi-square analysis showed no statistically significant difference between groups (χ²=0.00, p=1.00). Gender did not influence surgical selection.

**Table 2:** Gender distribution of patients in collagen and fat plug myringoplasty groups. Values are expressed as N (%). Chi-square test applied; results reported as χ²-value with p-value; p<0.05 considered statistically significant.

Gender	Collagen (n=23)	Fat (n=23)	χ²-value	p-value
Male	12 (52.2%)	12 (52.2%)	0	1
Female	11 (47.8%)	11 (47.8%)	0	1

Side of perforation

In the collagen group, 14 patients (60.9%) had left-sided perforations and nine (39.1%) had right-sided perforations. In the fat group, 11 patients (47.8%) had left-sided and 12 (52.2%) had right-sided perforations (χ²=0.35, p=0.55) (Table 3).

**Table 3:** Side of tympanic membrane perforation among collagen and fat plug myringoplasty groups. Values are expressed as N (%). Chi-square test applied; results reported as χ²-value with p-value; p<0.05 considered statistically significant.

Side	Collagen (n=23)	Fat (n=23)	χ²-value	p-value
Left	14 (60.9%)	11 (47.8%)	0.35	0.55
Right	9 (39.1%)	12 (52.2%)	0.35	0.55

Perforation quadrant and etiology

In the collagen group, perforations were posterosuperior in seven (30.4%), anterosuperior in two (8.7%), anteroinferior in seven (30.4%), and posteroinferior in seven (30.4%) patients. In the fat group, perforations were posterosuperior in eight (34.8%), anterosuperior in none (0%), anteroinferior in seven (30.4%), and posteroinferior in eight (34.8%) patients (χ²=2.12, p=0.54). No statistically significant difference was observed (Table 4).

**Table 4:** Distribution of tympanic membrane perforation quadrants among collagen and fat plug myringoplasty groups. Values are expressed as N (%). Chi-square test applied; results reported as χ²-value with p-value; p<0.05 considered statistically significant.

Quadrant	Collagen (n=23)	Fat (n=23)	χ²-value	p-value
Posterosuperior	7 (30.4%)	8 (34.8%)	2.19	0.54
Anterosuperior	2 (8.7%)	0 (0%)	2.19	0.54
Anteroinferior	7 (30.4%)	7 (30.4%)	2.19	0.54
Posteroinferior	7 (30.4%)	8 (34.8%)	2.19	0.54

In the collagen group, perforations were infective in 11 (47.8%), traumatic in five (21.7%), and post-myringoplasty in seven (30.4%) patients. In the fat group, perforations were infective in 11 (47.8%), traumatic in five (21.7%), and post-myringoplasty in seven (30.4%) patients (χ²=0.06, p=0.97). No statistically significant difference was observed (Table 5).

**Table 5:** Etiology of tympanic membrane perforation among collagen and fat plug myringoplasty groups. Values are expressed as N (%). Chi-square test applied; results reported as χ²-value with p-value; p<0.05 considered statistically significant.

Etiology	Collagen (n=23)	Fat (n=23)	χ²-value	p-value
Infective	11 (47.8%)	11 (47.8%)	0.07	0.97
Traumatic	5 (21.7%)	5 (21.7%)	0.07	0.97
Post-myringoplasty	7 (30.4%)	7 (30.4%)	0.07	0.97

Hearing outcomes (air-bone gap)

The mean preoperative air-bone gap was 24.35±6.76 dB in the collagen group and 25.78±6.30 dB in the fat group (t=0.82, p=0.426). At one month, it improved to 15.74±6.76 dB in the collagen group and 13.83±6.46 dB in the fat group (t=1.00, p=0.331). At two months, it further improved to 10.83±5.40 dB in collagen and 9.65±5.90 dB in fat (t=0.54, p=0.592). Both groups showed highly significant intra-group improvement from preoperative values (paired t-test, p<0.0001). Both groups showed highly significant intra-group improvement (p<0.0001). No statistically significant difference was noted between techniques at any time point (Pre-op: t=0.81, p=0.426; one month: t=0.98, p=0.331; two months: t=0.54, p=0.592) (Table 6).

**Table 6:** Preoperative and postoperative mean air-bone gap (ABG) in collagen and fat plug myringoplasty groups. Values expressed as mean ± SD. Independent t-test applied; results reported as t-value with p-value; p<0.05 considered statistically significant. Intra-group comparisons performed using paired t-test.

Time Point	Collagen (n=23) (Mean±SD)	Fat (n=23) (Mean±SD)	t-value	p-value
Pre-op	24.35±6.76	25.78±6.30	0.81	0.426
One month post-op	15.74±6.76	13.83±6.46	0.98	0.331
Two months post-op	10.83±5.40	9.65±5.90	0.54	0.592

Surgical success and failure rates

In the collagen group, graft success was achieved in 18 patients (78.3%) and failure in five (21.7%). In the fat group, graft success was achieved in 20 patients (87.0%) and failure in three (13.0%) (χ²=0.15, p=0.697). No statistically significant difference in success rates was observed between the two techniques (Table 7).

**Table 7:** Graft success rate in collagen and fat plug myringoplasty groups. Values are expressed as N (%). Chi-square test applied; results reported as χ²-value with p-value; p<0.05 considered statistically significant.

Outcome	Collagen (n=23)	Fat (n=23)	χ²-value	p-value
Success	18 (78.3%)	20 (87.0%)	0.15	0.697
Failure	5 (21.7%)	3 (13.0%)	0.15	0.697

By etiology, the success rate for infective perforations was 86.6% in the collagen group compared with 82.3% in the fat group. In traumatic perforations, collagen achieved a 60% success rate, while the fat group achieved 100%. Similarly, for post-myringoplasty cases, the success rate was 60% in the collagen group and 100% in the fat group (χ²=2.35, p=0.31). No statistically significant difference was observed between groups based on etiology (Table 8). Failure analysis revealed that infection was the predominant cause in both groups. Collagen failures included otomycosis and persistent discharge, while fat graft failures were evenly distributed among otomycosis, discharge, and extrusion.

**Table 8:** Success rates stratified by etiology in collagen and fat plug myringoplasty groups. Values expressed as percentage (%). Chi-square test applied; p<0.05 considered statistically significant.

Etiology	Collagen Success (%)	Fat Success (%)	χ²-value	p-value
Infective	86.6	82.3	0.82	0.36
Traumatic	60	100	0.82	0.36
Post-myringoplasty	60	100	0.82	0.36

Graft healing and closure rates

At day 7, healing was observed in 16 patients (69.6%) in the collagen group versus 15 (65.2%) in the fat group, while discharge was seen in seven (30.4%) versus eight (34.8%). At day 14, closure was achieved in 16 (69.6%) versus 17 (73.9%), with persistent perforation in three (13.0%) versus two (8.7%). At one month, closure was seen in 18 (78.3%) versus 20 (87.0%). At two months, closure was achieved in 18 (78.3%) versus 21 (91.3%) (χ²= 1.23, p=0.54). No statistically significant difference in healing and closure rates was observed between the two groups (Table 9).

**Table 9:** Postoperative healing and closure rates in collagen and fat plug myringoplasty groups at different time points. Values are expressed as N (%). Chi-square test applied; results reported as χ²-value with p-value; p<0.05 considered statistically significant.

Time Point	Collagen (n=23)	Fat (n=23)	χ²-value	p-value
Day 7 - Dry/Healing	16 (69.6%)	15 (65.2%)	1.12	0.29
Day 7 - Wet/Discharge	7 (30.4%)	8 (34.8%	1.12	0.29
Day 14 - Complete closure	16 (69.6%)	17 (73.9%)	1.12	0.29
Day 14 - Persistent perforation	3 (13%)	2 (8.7%)	1.12	0.29
1 Month - Closure	18 (78.3%)	20 (87.0%)	1.12	0.29
2 Months - Closure	18 (78.3%)	21 (91.3%)	1.12	0.29

Preoperative and postoperative photographs

**Figure 1:**
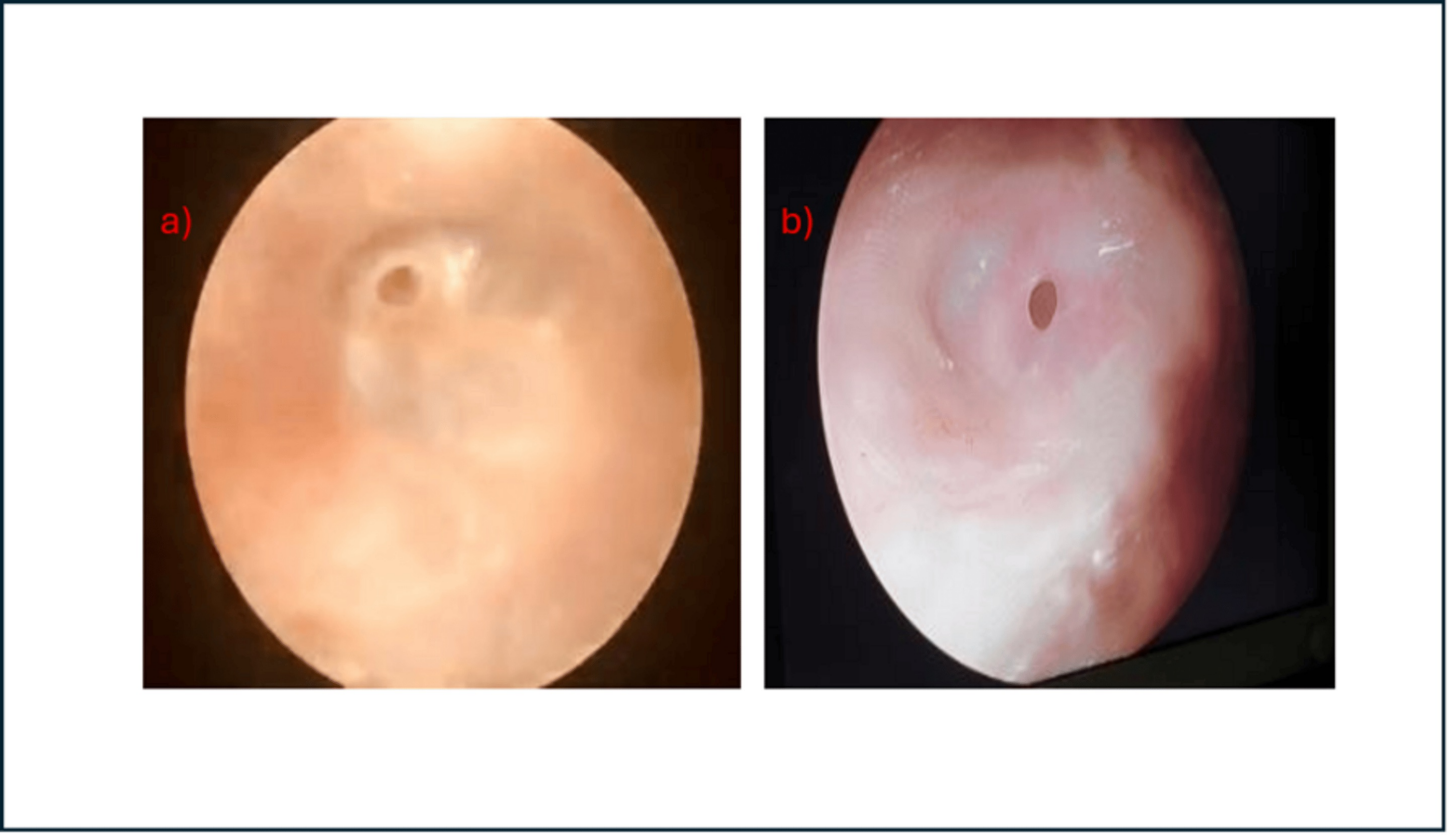
Preoperative tympanic membrane perforation. (a) Tympanic membrane perforation planned for collagen myringoplasty. (b) Tympanic membrane perforation planned for fat myringoplasty.

**Figure 2:**
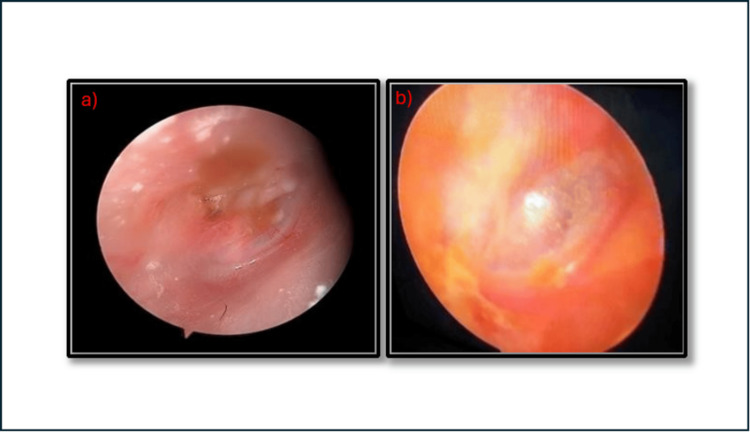
Postoperative tympanic membrane closure. (a) Tympanic membrane after collagen myringoplasty showing intact graft. (b) Tympanic membrane after fat myringoplasty showing successful closure.

Preoperative and postoperative ear photographs are depicted in Figures 1a, 1b and 2a, 2b, respectively. Figure 1 illustrates the preoperative tympanic membrane perforations, with Figure 1a showing a perforation planned for collagen myringoplasty and Figure 1b showing a perforation planned for fat myringoplasty.

Figure 2 demonstrates the postoperative outcomes, where Figure 2a shows an intact graft following collagen myringoplasty and Figure 2b shows successful closure of the perforation after fat myringoplasty.

## Discussion

Minor TM perforations are commonly encountered in ENT practice and can result from infections, trauma, or prior surgeries. These perforations often lead to conductive hearing loss, recurrent otorrhea, and chronic middle ear inflammation if left untreated. While spontaneous healing is possible for small perforations, surgical repair remains the standard for persistent defects. Among the various techniques, minimally invasive office-based methods using biological grafts such as collagen patches or autologous fat plugs have gained popularity due to their simplicity, cost-effectiveness, and reduced morbidity compared to traditional tympanoplasty [12-14].

In our study, patient demographics, including age, gender, and side of perforation, were comparable between the collagen and fat myringoplasty groups, with no statistically significant differences (p>0.05), consistent with previous reports indicating that these factors do not significantly influence surgical choice or outcomes [15,16]. Perforation site distribution was also similar, with most cases involving posterior and inferior quadrants, supporting earlier findings that small central perforations across different quadrants can be effectively treated using either technique [17].

Etiologically, the majority of perforations were infective in origin, followed by traumatic and post-myringoplasty cases, with no significant differences between groups (p=0.97). Baseline audiometry showed comparable preoperative air-bone gaps in both groups, confirming similar starting levels of hearing impairment. Postoperatively, both techniques led to significant improvement in hearing over the two-month follow-up (p<0.0001), with reductions in air-bone gap from approximately 24-26 dB preoperatively to 9-11 dB at two months. Intergroup comparisons revealed no statistically significant differences, indicating comparable efficacy of both graft materials in improving auditory function [12,18,19].

The overall surgical success rate was high for both groups - 87% in the fat plug myringoplasty group and 78.3% in the collagen patch myringoplasty - without a statistically significant difference (p=0.697). However, stratification by etiology revealed that fat myringoplasty achieved 100% success in traumatic and recurrent perforations, while collagen patch success was only 60% in these categories, highlighting the superior adaptability of autologous fat in challenging cases [14,20]. Failure analysis indicated that collagen graft failures were predominantly due to infection, particularly otomycosis and persistent discharge, as well as occasional graft extrusion. Fat graft failures were rarer and included infection, discharge, or mechanical extrusion, underscoring the generally better integration of autologous tissue [12,14].

Graft healing progressed favourably in both groups. By postoperative day 7, dry/healing grafts were observed in 69.6% of the collagen group and 65.2% of the fat group. By one month, closure rates improved to 78.3% and 87%, respectively, and by two months, collagen achieved 78.3% while fat reached 91.3%. These findings suggest that although early healing is similar, fat plugs demonstrate superior long-term closure rates, likely due to enhanced biocompatibility and resistance to extrusion or infection [13,14,21].

Overall, both collagen patch and fat plug techniques are effective for small TM perforation repair, improving hearing and facilitating reliable graft uptake. However, fat myringoplasty demonstrates distinct advantages in long-term healing and in managing traumatic or recurrent perforations, making it a preferred option in complex cases.

Limitations of the study

The present study has certain limitations. It was conducted at a single tertiary care center with a relatively small sample size of 46 patients, which may restrict the generalizability of the findings. The follow-up period was limited to two months, and therefore long-term outcomes such as delayed graft failures, late infections, or progressive audiological changes could not be evaluated. In addition, potential confounders such as Eustachian tube function, middle ear mucosal status, and individual variations in immune response were not comprehensively assessed. Future studies with larger, multicentric cohorts and extended follow-up are warranted to validate and expand upon these findings.

## Conclusions

In conclusion, both collagen patch and fat plug myringoplasty are effective and safe techniques for repairing small tympanic membrane perforations, resulting in significant improvement in hearing and favorable graft uptake. Fat plug myringoplasty, however, demonstrated superior long-term closure rates, particularly beyond the first month, and showed 100% success in traumatic and recurrent perforations, highlighting its reliability in more challenging cases. While both techniques are vulnerable to postoperative infection and occasional graft extrusion, fat plugs appear more resilient and better integrated, likely due to their autologous nature. Overall, these findings support the preferential use of fat plug myringoplasty for small central perforations, especially in scenarios where maximal graft uptake and long-term stability are critical, while collagen patches remain a viable alternative in routine cases.
